# Home Difficulties Experienced by Male Firefighters in South Korea: A Qualitative Study on Work–Family Conflict

**DOI:** 10.3390/healthcare13182300

**Published:** 2025-09-14

**Authors:** Nayoon Lee, Hyun-Ju Lee

**Affiliations:** 1 College of Nursing, Dong-A University, 32 Daesingongwon-ro, Seo-gu, Busan 49201, Republic of Korea; 095750@dau.ac.kr; 2 College of Nursing, Catholic University of Pusan, 57 Oryundae-ro, Geumjung-gu, Busan 46252, Republic of Korea

**Keywords:** firefighters, family support, work–family conflict, qualitative research, phenomenology

## Abstract

**Background/Objectives**: Family is a key protective factor for firefighters’ mental health, yet the home-related challenges of firefighting’s occupational demands remain underexplored in South Korea. This study aimed to establish an initial understanding of these challenges by conducting in-depth interviews with male firefighters and analyzing their lived experiences using a phenomenological approach. **Methods**: Twenty-nine married male firefighters (mean age = 43.4 years, range = 31–55) affiliated with the Busan Fire Department participated in individual telephone interviews between April and July 2022 during the COVID-19 pandemic. Data were analyzed using Giorgi’s descriptive phenomenological method, with NVivo Pro 12.0 employed for coding and organization. **Results**: Six themes were identified: (1) acting as an emergency commander at home, (2) reinterpreting traumatic experiences after marriage, (3) physical and emotional exhaustion from irregular schedules, (4) being national heroes misunderstood by families, (5) guilt-ridden and indebted Superman, and (6) striving to be Superman at home as well. These themes reflected the overarching meaning of a “lonely breadwinner struggling to be Superman both at work and home.” Participants described hyperarousal, emotional withdrawal, strained relationships, guilt over missed family events, and compensatory overextension. Distress was often concealed to protect families, but this limited emotional support. **Conclusions**: Korean male firefighters face significant work–family conflict shaped by cultural and occupational factors. These findings highlight the need for family-centered counseling, psychoeducation, and organizational interventions. Policy measures such as guaranteed rest after shifts, couple-based communication programs, and resilience-building initiatives are recommended to strengthen families as vital sources of psychological resilience.

## 1. Introduction

Firefighters are routinely exposed to traumatic events while performing duties such as fire suppression, emergency medical services, and rescue operations, making them particularly vulnerable to post-traumatic stress disorder (PTSD) [1,2]. The high-stress nature of shift work and constant operational pressure also places firefighters at greater risk than the general population for mental health conditions, including sleep disturbances [3], depression and anxiety [4], and alcohol use disorders [5].

The occupational demands of firefighting pose inherent risks to mental well-being. These demands are difficult to modify because irregular shifts, emergency mobilization, and repeated traumatic exposures are unavoidable aspects of the job. Therefore, it is essential to identify protective factors that can mitigate adverse mental health outcomes. Among these, social support has emerged as a crucial buffer, shown to alleviate burnout [6,7], PTSD [7], and depression [8], while enhancing job satisfaction, quality of life, and post-traumatic growth.

A notable study examining different sources of social support among firefighters [9] found that while peer and organizational support had limited influence on encouraging self-disclosure of traumatic experiences, high levels of family support significantly promoted self-disclosure and, in turn, deliberate rumination—ultimately facilitating post-traumatic growth. Additionally, firefighters reporting lower satisfaction in their romantic relationships experienced a greater burden of mental health symptoms [10]. These findings underscore the vital role of family support in the mental health recovery process for firefighters.

In South Korea, firefighters typically work a 24 h on/48 h off rotating schedule, which requires long overnight shifts and frequent emergency mobilization. Their duties are broadly divided into fire suppression, rescue, emergency medical services, fire investigation, situation room operations, administrative tasks, and training programs. As of 31 December 2024, there were 66,802 career firefighters nationwide, including 6935 women (10.4%), indicating that the workforce remains male-dominated [11]. The fire service also retains a paramilitary organizational culture, with command-oriented communication and sensitivity to rank, reflecting the life-or-death nature of the work.

However, firefighters are often subject to nonstandard work schedules—such as 24 h shifts, night and weekend duty, and emergency call-ins—which frequently disrupt personal and family life [12,13]. These occupational patterns have been associated with lower marital satisfaction, increased family conflict [14,15], and elevated divorce rates [16]. In particular, such work conditions can erode family support, intensify work–family conflict, and ultimately contribute to burnout [17,18]. According to the World Health Organization [19], burnout is a syndrome resulting from chronic workplace stress that has not been successfully managed, and it is characterized by exhaustion, mental distance or cynicism, and reduced professional efficacy. Burnout, in turn, has been linked to reduced adherence to safety behaviors, such as compliance with personal protective equipment protocols [20,21]. The deterioration of family support and escalation of work–family conflict, therefore, not only affect individual firefighters but may also pose broader risks to organizational and public safety, highlighting the need for social and policy-level interventions.

Although previous studies have emphasized the need for intervention programs that strengthen family support as a protective factor [9,10], no such programs have been developed in South Korea, and foundational research remains lacking. Furthermore, no qualitative studies have explored how firefighters perceive and experience family-related support or conflict.

While some international qualitative studies have examined family conflict among firefighters [22,23,24,25], their contexts vary considerably, including studies from Ireland, the United States, Canada, Australia, and the United Kingdom. However, cultural and structural differences—such as Confucian gender-role expectations, collectivistic norms, and hierarchical organizational culture—limit the direct applicability of these findings to South Korea and uniquely shape how Korean male firefighters experience work–family conflict. In particular, gender-role expectations often position men as primary providers and women as caregivers [26]. Moreover, Confucian collectivistic values emphasize sacrifice and duty to family and society [27], while the hierarchical organizational culture of the fire service fosters authority-driven interactions that may spill over into family life [24,28]. Therefore, there is a clear need to explore the lived experiences of Korean firefighters, particularly within male firefighter–female spouse dynamics, to understand how family support is shaped and challenged in the context of Korean cultural and organizational norms.

In South Korea, 89.6% of firefighters are male [11], reflecting a highly male-dominated workforce. To date, no studies—qualitative or quantitative—have examined work–family conflict or home-related difficulties among Korean firefighters and their families. Given this research gap, our study focused exclusively on male firefighters to establish an initial empirical foundation.

This study thus aims to explore, through qualitative methods, the family-related challenges faced by male firefighters in South Korea owing to their occupational demands. The findings are intended to inform the development of family-based counseling and support programs, including organizational policy improvements. Ultimately, this research seeks to promote firefighters’ mental health and contribute to the safety of local communities and the broader public.

While exploring lived experiences is the primary objective, it is equally important to interpret these findings within broader theoretical frameworks. Given the limited empirical evidence in the Korean context, theoretical framing is essential to strengthen the conceptual contribution of this study. The Job Demands–Resources (JD–R) model [29] and the Conservation of Resources (COR) theory [30] offer valuable perspectives for understanding firefighters’ work–family conflict. The JD–R model posits that high job demands, when not balanced by sufficient job or personal resources—including social resources such as family support—lead to strain and burnout. COR theory further suggests that individuals strive to acquire, protect, and maintain resources, and stress occurs when resources are threatened or depleted. Applied to firefighters, the intense occupational demands of shift work, traumatic exposure, and organizational pressures consume psychological and social resources, thereby limiting the capacity to meet family expectations and increasing conflict at home. By situating our findings within these frameworks, the present study seeks not only to describe lived experiences but also to highlight underlying mechanisms that explain why and how firefighters’ occupational stressors disrupt family life.

This study aimed to gain an in-depth understanding of the challenges firefighters face in their family lives and to explore the underlying nature of these experiences. Specifically, through individual in-depth interviews analyzed using Giorgi’s descriptive phenomenological method [31], it seeks to investigate and characterize the various difficulties encountered at home owing to the unique demands and occupational characteristics of firefighting. This study’s central research question is: “What challenges do male firefighters experience at home because of their occupational demands?”

## 2. Materials and Methods

### 2.1. Study Design

This qualitative study employed individual in-depth interviews to gain a deeper understanding of the challenges male firefighters face in their family lives due to the nature of their work.

### 2.2. Participants and Setting

Participants were male firefighters affiliated with the Busan Fire Department who had marital experience (currently married, divorced, or separated) and who self-identified as having experienced family-related difficulties arising from occupational demands. Participation was voluntary, and self-identification was used as the basis for inclusion. A combination of convenience and snowball sampling strategies was employed. After IRB approval, a recruitment notice was posted, and participation was voluntary. In addition, participants were encouraged to recommend colleagues who might be eligible, to enhance diversity in the sample. Firefighters without marital experience or who did not report family-related difficulties were excluded. In the South Korean cultural context, marital life is strongly associated with unique social and familial responsibilities that differ from non-marital partnerships. For this reason, we included firefighters who had marital experience (currently married, divorced, or separated) rather than restricting the sample to those who were presently married. This approach also aimed to capture the experiences of firefighters whose work–family conflict had contributed to marital dissolution.

Busan, a metropolitan city in South Korea, features a complex urban landscape that includes coastal areas, mountains, high-rise buildings, industrial zones, ports, and tourist attractions. Consequently, firefighters in this region are frequently exposed to a wide range of emergencies and high work intensity. Given these characteristics, this population was deemed appropriate for exploring the work-related family challenges.

After the study’s purpose and methodology were explained to the public official in charge of health and safety at the Busan Fire Department, permission was obtained to recruit participants. A recruitment notice was posted on the department’s internal bulletin board and social media channels. Ultimately, 29 firefighters volunteered and participated in the study.

All participants were male firefighters, with a mean age of 43.4 years (range: 31–55). The average duration of marriage was 11.4 years (range: 2–15), and the average number of children was 1.8 (range: 0–4). Although the inclusion criteria allowed firefighters with marital experience (currently married, divorced, or separated), in practice, all participants were currently married at the time of the study. Regarding occupational roles, 14 participants worked in fire suppression, five in emergency medical services, four in administration, three in driving, and one in rescue, control center operations, and fire investigation.

### 2.3. Data Collection and Procedure

The original research plan involved conducting focus group interviews. However, one day before the first scheduled session, social distancing guidelines for firefighters were strengthened to prevent the spread of COVID-19. Consequently, the data collection method was changed to include individual telephone interviews. Data were collected from April to July 2022.

Each first-round interview lasted approximately 60 min. For the two participants with ambiguous initial responses, follow-up interviews were conducted via mobile phone for approximately 10 min. Data collection was concluded when no new information emerged regarding firefighters’ difficulties at home, indicating theoretical saturation.

The interviews commenced with an open-ended question designed to elicit participants’ lived experiences:

*“Can you describe the difficulties you face at home owing to your work as a firefighter?”*

*Additional semi-structured prompts were employed* *to further explore the depth and complexity of their experiences and support the development of emergent themes, including: “In what ways does your role as a firefighter impact your family life?* *”, What types of family conflict have arisen because of your job?”* *, and “How do you manage work-related stress at home?”*

To prevent data distortion or loss during the remote interviews, all audio recordings were transcribed verbatim by co-researchers who reviewed each recording multiple times. Special attention was given to nonverbal cues, such as pauses, tone, speed, inflection, and emotional expression. Notes taken during the interviews were reviewed to ensure the accuracy of the transcription.

Data collection and analysis were conducted simultaneously using an iterative process. Each new transcript was compared with previous transcripts to identify emerging patterns. No new themes were identified from the 28th interview onward, and data saturation was confirmed.

### 2.4. Data Analysis

Data were analyzed using Giorgi’s descriptive phenomenological method [31] with NVivo Pro 12.0 (QSR International Pty Ltd., Melbourne, Australia) as a supplementary tool to organize and code meaning units efficiently. The analysis followed the following sequential steps:

First, the researchers read all the interview transcripts multiple times to understand the participants’ experiences. Second, the transcripts were imported into NVivo Pro 12.0, and meaning units were identified by closely rereading the participants’ statements. Regardless of the differences in wording, expressions that captured the essence of firefighters’ family-related difficulties were extracted.

Third, the identified meaning units were conceptualized and transformed into academically relevant expressions. Before this step, all meaning units were shared among the research teams, and the researchers discussed and refined them through three iterative meetings. The final units were then integrated into constituent elements and systematically categorized. Finally, the essential structure of the phenomenon was derived by verifying that each element was consistently represented across participants’ experiences and synthesizing the findings into a comprehensive and cohesive description.

We also made deliberate efforts to identify and record negative or deviant cases in order to capture heterogeneity in participants’ experiences and to minimize the risk of overgeneralization.

### 2.5. Ethical Considerations

Before the commencement of this study, ethical approval was obtained from the Institutional Review Board of the principal investigator’s affiliated institution (CUPIRB-2021-039-01). Per the principles outlined in the Declaration of Helsinki, all participants were fully informed about the study’s purpose and procedures and the safeguards in place to ensure anonymity and confidentiality.

Written informed consent was obtained from each participant in duplicate; the researcher retained one copy, and the other was given to the participant. The participants were clearly informed of their right to withdraw from the study at any time without any negative consequences.

All personal identifiers were removed during the transcription and reporting to protect anonymity. The signed consent forms will be securely stored for three years after the study’s completion and then destroyed per institutional policy. Participants received a small gift voucher upon completing the interview as a token of appreciation.

### 2.6. Rigor

To ensure the trustworthiness of this study, we applied Lincoln and Guba’s evaluation criteria for qualitative research, namely credibility, transferability, dependability, and confirmability [32].

To enhance credibility, interviews were conducted to minimize bias and preconceptions. Participants’ narratives were listened to attentively, and verbatim transcriptions were completed on the same day to preserve vivid details and emotional nuances. Additionally, three participants were invited to review the categorized concepts to verify whether the findings accurately reflected their intended meanings.

For transferability, the final results were presented to three firefighters who had not participated in the study to determine whether the findings resonated with their experiences and perceptions.

To ensure dependability, meaning units were compared and integrated according to Giorgi’s descriptive phenomenological method [31]. Any research discrepancies were addressed through discussion until a consensus was reached.

To establish confirmability, researchers acknowledged their own reflexivity and positionality. The first author’s extensive counseling experience with firefighters and the corresponding author’s qualitative research expertise and personal familiarity with firefighters’ family life were considered as potential influences during interpretation. To minimize this risk, peer debriefing and bracketing were employed to ensure that findings reflected participants’ lived experiences rather than researchers’ preconceptions.

## 3. Results

A total of 31 telephone-based in-depth interviews were conducted, and six major themes and 23 subthemes were identified using phenomenological analysis (Table 1). The essential structure of the phenomenon, as revealed through all thematic categories, was captured in the phrase: “Lonely breadwinner struggling to be Superman both at work and home”.

These findings clearly depict the lived experiences of male firefighters in Korea regarding the difficulties they face with their families. Even at home, where they should be able to rest, firefighters reported being unable to relax and maintain a heightened sense of alertness as if they were still commanding an emergency. After marriage, they were more emotionally affected by traumatic incidents than those who were single. Such experiences made them reassign meaning to these events, including a deeper recognition of the family’s values.

Participants also expressed physical exhaustion due to irregular and unpredictable work schedules, including feelings of guilt for disrupting or missing family routines. Although firefighters are perceived as national heroes by society, many firefighters experience emotional conflict at home when their family members lack an understanding of the demands of their work, resulting in insufficient emotional support and relationship strain.

They frequently felt guilty and indebted to their families due to night shifts and emergency duties. They often overextend themselves at home to compensate for their absence and emotional distance, leading to further emotional fatigue. Finally, out of concern that their job might negatively impact family life, they constantly tried to reassure their families. They strove to maintain the image of a strong and flawless figure, struggling to fulfill the role of a “Superman” at work and home.

### 3.1. Acting as an Emergency Commander at Home

This theme illustrates how firefighters remain in heightened physiological and psychological arousal after returning home from their high-stress duties at the fire station. This sustained tension often manifests as irritability and an authoritative communication style, leading to conflict within the family. For example, sleep disturbances stemming from hypervigilance frequently disrupt not only firefighters’ rest but also that of their spouses.

#### 3.1.1. Heightened Arousal

Participants reported that their elevated arousal levels did not subside upon returning home. In particular, they became overly sensitive to safety-related issues, which often led to friction among their family members.

*“When my kids were about to run across the crosswalk, scenes from accident sites flashed through my mind, and I yelled at them more than necessary. My wife and children did not understand why I was so upset. I used to be calm, but now I get angry and irritable more often.”*

(Participant 9)

*“Even on my day off, I take my phone into the bathroom in case I am called for emergency duty. I can never fully relax, even when I am off duty.”*

(Participant 15)

#### 3.1.2. Sleep Disturbances Affecting the Spouse

Because of their occupational conditioning to respond immediately to alarms, firefighters have reported becoming overly sensitive to even minor sounds at home. This hypervigilance often disrupts the sleep of their spouses.

*“I jump up at the slightest noise at night, which causes my wife to lose sleep too. Eventually, she told me to sleep in another room. However, now that we have been sleeping separately for so long, it feels awkward to suggest sharing a bed again.”*

(Participant 27)

#### 3.1.3. Authoritative Communication Leading to Conflict

Participants described adopting a commanding communication style at home, a habit formed in the rigid hierarchical environment of a fire station. This tone often results in tension and misunderstanding among family members.

*“When my kids ask, ‘Why?’ I catch myself saying, ‘Just do as you are told,’ as if I am disciplining them in the field. My wife snapped back, ‘I am not your subordinate.’”*

(Participant 11)

### 3.2. Reinterpreting Traumatic Experiences After Marriage

This theme captures how firefighters’ experiences of traumatic events undergo a profound shift after marriage and having children. Participants reported increased preoccupation with family safety and heightened emotional sensitivity, particularly in response to incidents involving infants or young children. These events often result in secondary trauma and deepen family appreciation. At the same time, variations were evident: while many participants—especially those with younger children—reported intensified marital conflict and emotional strain, a few described strengthened family cohesion and renewed appreciation, underscoring heterogeneity in how family stage shapes responses to occupational stress.

#### 3.2.1. Irritability or Withdrawal at Home After Traumatic Incidents

Participants described how emotional distress stemming from traumatic incidents frequently led to irritability or emotional withdrawal at home, which often triggered conflicts with their spouses.

*“After I had a family, accident scenes started to hit me harder. On such days, I either withdraw into silence or become irritable at home. If I am irritable, my wife gets angry; if I stay silent, she also gets upset…”*

(Participant 22)

#### 3.2.2. Excessive Safety Warnings Toward Family

Following traumatic experiences on duty, many participants reported becoming excessively cautious about their safety at home. They often gave their families repeated warnings or unsolicited advice, which became a source of frustration for family members.

*“After returning from a fire scene, I cannot stop telling them to change the power strip or unplug the cords—it is nonstop safety preaching.”*

(Participant 24)

*“After seeing a bus crash, I would not let my kids go on school trips. They were very frustrated.”*

(Participant 1)

#### 3.2.3. Increased Appreciation for Family

Exposure to tragic events during emergency response led participants to feel a renewed and intensified sense of gratitude toward their families, an emotional shift rarely felt before marriage.

*“After I return from a serious incident, I cannot help but think, ‘You never know what will happen tomorrow. I have to give my best to my family today.’”*

(Participant 21)

#### 3.2.4. Greater Emotional Involvement in Incidents After Having Children

Many participants shared that, after becoming parents, they found it increasingly challenging to handle incidents involving their children. This emotional connection reminds them of their children, heightening the psychological burden of such events.

*“When I see a child around my kids’ age involved in an accident, I cannot help but think of my own child. My hands shake, I tear up, and it becomes emotionally unbearable.”*

(Participant 22)

### 3.3. Physical and Emotional Exhaustion Due to Irregular Work Schedules

This theme highlights the cumulative experience of firefighters strained by their irregular work hours. During night shifts, they reported emotional distress from worrying about their families, and upon returning home, they experienced physical exhaustion. The 24 h shift structure, in particular, intensified conflict with spouses, especially concerning childcare responsibilities and missed family events due to sudden emergency mobilization.

#### 3.3.1. Inadequate Rest After Night Shifts

Participants reported difficulty obtaining sufficient rest after night duty, particularly because of disruptions from young children at home. Over time, this leads to chronic fatigue and compromises physical recovery.

*“When I come home after a night shift, my kids knock on the bedroom door asking to play, or they make noise. I end up not sleeping, and my eyes are always bloodshot.”*

(Participant 1)

#### 3.3.2. Anxiety About Family’s Safety During Night Shifts

On duty, firefighters expressed ongoing anxiety about the safety of their spouses and children, stating that their thoughts frequently returned home despite being at work.

*“During my 24-h shift, I cannot relax because I worry about my wife and kids being alone at home.”*

(Participant 4)

#### 3.3.3. Heightened Conflict with Spouse over Childcare

Many of the participants reported that their extended duty hours placed a full burden on their spouses. Upon returning home exhausted, they often respond irritably to their partners’ expressions of frustration, leading to frequent conflict.

*“If one of our two kids gets sick, my wife has no one to take the child to the hospital or stay home with the other. She is completely worn out, and I come home totally exhausted. We end up fighting all the time.”*

(Participant 9)

#### 3.3.4. Sacrificing Personal Plans Due to Emergency Mobilization

Participants also shared that the unpredictability of emergency mobilization, especially during the summer with typhoons and heavy rainfall, led them to give up their personal and family plans.

*“During the summer, with all the typhoon and heavy rain warnings, I never know when I will be called in. Therefore, I give up summer vacations and even a simple beer in the evening.”*

(Participant 17)

### 3.4. National Heroes Misunderstood by Their Families

This theme highlights the emotional burden that firefighters carry when their families do not fully understand or acknowledge the demands of their work. Due to limited opportunities for family education about firefighting duties and firefighters’ tendency to downplay the dangers of their jobs to protect their loved ones, family members often misinterpret or underestimate their workload. This lack of awareness results in insufficient emotional support and limited opportunities for proper rest at home.

#### 3.4.1. Families Consider Standby Time as Rest Time

Participants often minimized the frequency or severity of emergency calls to ease their families’ concerns. However, this well-intentioned reassurance led to misunderstandings, as family members came to view standby time as equivalent to rest and expected firefighters to be fully available for domestic responsibilities upon returning home.

*“Everyone knows firefighting is dangerous; thus, my wife often worried about me. To ease her anxiety, I told her that we rarely get dispatched and that nothing serious happens. I even told her there were no calls last night. Then she assumes I have been resting all night and dumps all the housework and childcare on me.”*

(Participant 22)

#### 3.4.2. Lack of Recognition for Post-Night-Shift Recovery Time

Firefighters reported that the rest days following night shifts were often not perceived as a legitimate recovery time. Instead, family members expect them to participate in outings or complete household tasks, preventing them from physically or mentally recuperating.

*“The next day off is meant for rest after a night shift. However, my family treats it as a day out; thus, the moment I get home, they urge me to go on outings. I cannot say no, and I cannot rest either.”*

(Participant 26)

#### 3.4.3. Complaints About Missing Important Family Events

Due to nonstandard and unpredictable work schedules, participants frequently missed major family occasions, such as holidays, birthdays, school events, and ceremonies. These absences often lead to disappointment and frustration among family members, contributing to recurring conflicts.

*“Given that I am always on duty during holidays, my wife and kids go to my parents’ place alone. We end up arguing every New Year’s.”*

(Participant 29)

*“I could not attend my child’s kindergarten sports day because of work. Both my child and wife were really disappointed.”*

(Participant 8)

### 3.5. Guilt-Ridden and Indebted Superman

This theme reveals how firefighters burdened by nonstandard work schedules and sacrifices made by their families often experience persistent guilt and indebtedness. Participants described a strong emotional obligation to compensate for these sacrifices, sometimes at the expense of their physical and emotional well-being.

#### 3.5.1. Guilt for Missing Important Family Moments

The participants expressed ongoing guilt over their inability to attend important family events because of their unpredictable duty schedules. Consequently, many gave up their personal hobbies or leisure activities to avoid further burdening their families.

*“My 24-h shifts definitely place a burden on my family. Therefore, I have given up my hobbies such as working out. I live each day with a constant sense of guilt.”*

(Participant 12)

#### 3.5.2. Feeling Indebted to Family Members Who Endure a Lot

Participants deeply empathized with the hardships their family members who were related to a firefighter endured. This emotional indebtedness motivated them to go above and beyond to “repay” what they perceived as a moral or relational debt.

*“We had planned a graduation trip for my child’s elementary school, but a colleague got injured and I had to cover his shift. We could not go. I have been ‘repaying’ that debt ever since—doing extra chores and childcare.”*

(Participant 21)

#### 3.5.3. Guilt Toward Family Members Who Worry About Their Safety

Given the inherently dangerous nature of firefighting, participants were keenly aware that their loved ones were constantly anxious about their safety, particularly during major incidents. This awareness evoked feelings of guilt and sorrow.

*“Every time there is a major fire, I know my family stays up all night checking the news to see if I am safe. They would not have to go through that if I had a regular office job. I always feel sorry for putting them through such stress.”*

(Participant 29)

#### 3.5.4. Overextending Themselves to Compensate Their Families

Many firefighters felt compelled to place their families’ needs before their own, even when physically or emotionally depleted, to compensate for their families’ sacrifices. This self-imposed obligation often results in exhaustion.

*“After a night shift, I try to take over all the childcare and housework so my wife can rest—because she suffers so much being married to a firefighter. Nonetheless, I am exhausted too, and one time I nodded off on a bench at the playground while my kid fell… I did not even notice. (laughs)”*

(Participant 23)

### 3.6. Endeavoring to Be Superman at Home as Well

This theme illustrates how firefighters make concerted efforts to shield their families from the adverse psychological and physical effects of work. Out of concern for their loved ones, they adjust their daily routines, suppress emotional distress, and strive to maintain a strong, dependable presence at home—continuing to play the role of “Superman” beyond the fire station.

#### 3.6.1. Self-Control to Prevent Firefighter Traits from Affecting Family Life

Participants knew that work-related traits, such as hypervigilance and authoritative communication styles, could cause tension at home. They described making conscious efforts to restrain these behaviors to preserve family harmony.

*“In summer, fan-related fires are common. Therefore, when I see the fan running in an empty room, I get really anxious—but I turn it off and hold back my words because I do not want to sound like I am nagging.”*

(Participant 12)

#### 3.6.2. Concern About Passing ‘Bad Things’ to the Family

Participants expressed fear that their occupational exposure, whether to traumatic events or hazardous materials, might harm their family members emotionally or physically. To prevent secondary traumatic stress, they avoided sharing distressing details and took extra measures to decontaminate themselves after the fire.

*“I had to recover a body after a fall. I was so disturbed, I wanted comfort from my wife—but I kept it to myself, worrying it might traumatize her. Nonetheless, my expression gave it away. She got upset, saying, ‘Why do you look like that? Did I do something wrong? Just tell me!’”*

(Participant 7)

*“After a fire, I am covered in soot and chemicals. Even though I shower and change at the station, I shower again at home and wash my work clothes separately before touching anyone.”*

(Participant 11)

#### 3.6.3. Sharing Trauma with Their Spouse

Some firefighters attempted to share their traumatic experiences with their spouses. Spousal responses to trauma disclosure varied considerably. Some firefighters felt comforted when their wives encouraged open communication, whereas others reported distress when their disclosures were dismissed. Several participants also later described negotiated adjustments with their spouses that helped reduce tensions, indicating adaptive family coping over time. When empathy was present, the support was deeply appreciated, but emotional disillusionment occurred when responses were dismissive.

*“When I opened up about a difficult call, my wife said, ‘You can always talk to me.’ That support was such a comfort.”*

(Participant 22)

*“One day, I could not eat or think straight after retrieving a severely mutilated body. When I told my wife I was unsure if I could keep doing this job, she said, ‘Quitting is not an option.’ That really hurt me.”*

(Participant 6)

#### 3.6.4. Adjusting Daily Life to Maintain Work–Family Balance

The participants reported intentionally adjusting their daily routines to balance their roles as firefighters, spouses, and parents. Although compromises, often from spouses, were necessary, they sought to resolve conflicts through mutual understanding and negotiation.

*“My wife and I both used to work shifts, and we hit our limits with childcare. She eventually switched to a daytime job. I feel guilty—it was a setback for her career.”*

(Participant 23)

*“As a uniformed officer, I used to obsess over promotions. Studying for exams and entertaining senior officers kept me away from my family. One day, my wife asked for a divorce. That is when I gave up chasing promotions.”*

(Participant 16)

*“After a night shift, we agreed that I get five hours of sleep—no questions asked. After that, I take over the chores and childcare. That agreement brought peace to our home.”*

(Participant 22)

#### 3.6.5. Increased Caution to Reassure the Family

Becoming a spouse or parent increased participants’ sense of responsibility, prompting them to be more cautious about their jobs. They strictly adhered to safety protocols and consciously worked to reassure their families about their well-being.

*“Since becoming the head of a family, I have become extra cautious during every task. My safety means peace of mind for my family.”*

(Participant 17)

**Figure 1:**
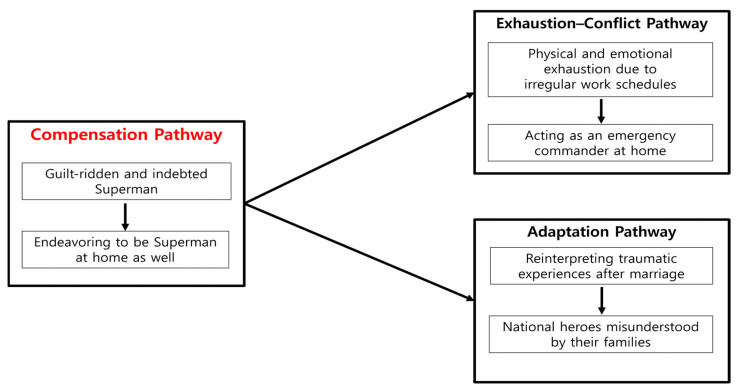
Thematic map of three pathways of home difficulties among Korean male firefighters.

Analysis of the findings revealed three overarching pathways that explain the home difficulties of Korean male firefighters: the Compensation Pathway, the Exhaustion–Conflict Pathway, and the Adaptation Pathway. Among these, the Compensation Pathway frequently acted as a central mechanism, as guilt and indebtedness often motivated firefighters to take on excessive responsibilities at home. This not only intensified exhaustion and conflict but also facilitated adaptive negotiations with spouses. To help readers visualize the interconnections among these themes, a thematic map is presented in Figure 1. The Compensation Pathway influences both the Exhaustion–Conflict and Adaptation Pathways.

## 4. Discussion

In the following sections, we summarize the key insights from each theme, interpret these findings in the context of existing literature, and discuss the practical and policy implications of the results.

Beyond describing lived experiences, the findings of this study can also be understood through established theoretical frameworks. The recurring “Superman role” and related family conflicts resonate with the JD–R model [29], which posits that high job demands without sufficient resources result in strain and spillover into home life. Similarly, the COR theory [30] explains how continuous resource depletion at work leads firefighters to withdraw emotionally or overextend themselves in family roles to compensate for losses. Situating our results within these frameworks underscores that the experiences reported by participants are not merely descriptive but also reflect broader theoretical mechanisms of stress, resource loss, and work–family conflict.

Although participants represented diverse occupational roles (e.g., suppression, EMS, administration), no clear role-based differences emerged, suggesting that the pervasive demands of irregular shifts and traumatic exposure cut across positions. By contrast, heterogeneity was evident in family contexts: spousal responses to trauma disclosure ranged from empathic support to dismissal, and some couples developed negotiated routines to reduce conflict. These variations illustrate how family-level resources can buffer or intensify job demands (JD–R) and how resource gains or losses within relationships shape outcomes (COR).

The Compensation Pathway emerged as the pivotal link between firefighters’ occupational stressors and their family lives. Guilt and indebtedness pushed firefighters to assume excessive responsibilities at home, fueling exhaustion and conflict while also prompting adaptive negotiations with spouses. Figure 1 illustrates how this central mechanism influenced the other two pathways.

### 4.1. Acting as an Emergency Commander at Home

The first theme revealed that firefighters often have a heightened arousal after returning from duty. This hyperarousal manifests as sleep disturbances, irritability, and authoritarian communication styles, which frequently trigger or escalate family conflicts, ultimately contributing to a vicious cycle of stress and relational discord.

These findings are well explained by the Spillover–Crossover Model proposed by Bakker et al. [33], which posits that stress experienced in the workplace “spills over” into the individual’s home life and eventually “crosses over” to affect the well-being of close family members. The present study illustrates both processes in line with previous research documenting the spillover of traumatic stress among first responders and its effects on family members [28,34].

Beyond the Spillover–Crossover Model [33], our findings can also be interpreted through the lens of the JD–R model [29] and COR theory [30], which provide a more comprehensive theoretical grounding. The JD–R model [29] explains how the combination of high occupational demands (e.g., irregular shifts, exposure to trauma, and emergency mobilization) and insufficient resources (e.g., rest time, organizational support, and family understanding) produces strain that spills over into the home domain. Similarly, COR theory [30] emphasizes that firefighters’ persistent loss of emotional and physical resources at work leaves them vulnerable to further depletion in family interactions, often resulting in withdrawal, irritability, or compensatory overextension. Integrating these frameworks into the interpretation of our results underscores that the work–family conflict experienced by Korean firefighters is not merely descriptive but reflects broader theoretical principles of resource dynamics and occupational stress.

Our findings also complement prior research on firefighter families in Western contexts [22,25] by illustrating how cultural and organizational features in Korea shape the mechanisms of work–family conflict. The expectation that men should shoulder the role of sole providers, combined with collectivistic norms of self-sacrifice and a hierarchical workplace culture, intensifies the pressure for firefighters to maintain authority and composure even at home. These cultural conditions suggest that firefighters’ family difficulties are not only the result of universal occupational demands but also reflect context-specific values and practices. Situating our results within these boundary conditions underscores the broader significance of this study, demonstrating that cultural context is critical for understanding the interplay between work and family in emergency service professions. For example, one prior study described a firefighter, after returning home from a traumatic call, as ‘a landmine waiting to go off,’ with his spouse forced to tiptoe around his unpredictable emotional state [28].

An emerging but underexplored pattern in our study is the identification of a specific pathway linking firefighters’ sleep disturbances to their spouses’ disrupted sleep, eventually leading to separate sleeping arrangements and diminished marital intimacy. This pattern, which has not been explicitly discussed in previous Western studies, underscores that the consequences of hyperarousal extend beyond the emotional and communicative domains, such as sleep quality and cohabitation, to impact the physical aspects of family life.

Moreover, we found that firefighters and their spouses frequently misattributed symptoms of hyperarousal, such as irritability and emotional detachment, to stable personality traits rather than recognizing them as potential indicators of PTSD. This misinterpretation may delay timely intervention and highlights the need for psychoeducation for firefighters and their families.

### 4.2. Reinterpreting Traumatic Experiences After Marriage

Following marriage and childbirth, firefighters appeared to internalize traumatic events more deeply, especially those involving children. Emotional intensification often leads to irritability or emotional withdrawal at home, which, in turn, contributes to conflicts with family members. These results are consistent with prior studies reporting that child-related fatalities are particularly associated with heightened trauma responses among first responders [35] and that overprotective behaviors aimed at family safety can create relational strain [36].

Importantly, our study reveals a contrasting but meaningful dynamic: the experience of post-traumatic growth. Several participants described how becoming a parent reframed their perception of traumatic incidents, prompting a renewed sense of life purpose and emotional clarity; for example, they expressed a commitment to cherish family relationships and live more intentionally. This finding supports the existing literature suggesting that trauma may also catalyze positive psychological transformation, helping individuals reevaluate personal values and strengthen their emotional bonds with loved ones [37].

### 4.3. Physical and Emotional Exhaustion Due to Irregular Work Schedules

Firefighters working 24 h shifts reported persistent fatigue due to inadequate rest following night duty. During working hours, they often experience anxiety about the safety of their spouses and children at home. Emergency mobilization frequently disrupts family plans, including vacations and events, leading to heightened conflict, particularly regarding childcare responsibilities and family schedule coordination.

Previous studies have shown that individuals with nonstandard work schedules are vulnerable to a range of physical and mental health problems, primarily due to disrupted sleep patterns and circadian rhythm disturbances [38,39]. This study builds on this literature by illuminating the specific mechanisms that hinder the rest and recovery of firefighters, particularly those with young children. Many participants described marital tension resulting from unequal childcare responsibilities during extended shifts. This finding aligns with previous qualitative research indicating that firefighter spouses often bear the burden of solo parenting [24].

Furthermore, previous studies have reported that male night-shift workers are nearly six times more likely to experience divorce or separation than daytime workers [40] and that shift-working parents of young children are more prone to depression and marital discord [41]. These findings underscore the significance of Theme 3 in illustrating the unique stress pathways through which irregular work schedules contribute to family conflicts in professions where night work is unavoidable.

### 4.4. National Heroes Misunderstood by Their Families

To protect their families from unnecessary worry, many firefighters reported intentionally minimizing the risks and fatigue associated with their jobs. These so-called “white lies” were aimed at emotional reassurance but inadvertently contributed to a distorted perception of their occupational reality. Consequently, family members often misinterpret standby duty as rest time and expect firefighters to assume full domestic responsibility upon returning home.

Additionally, repeated absences from important family milestones due to irregular work schedules have contributed to growing resentment and the gradual erosion of emotional support. Although firefighters are among the most respected public servants in South Korea [42], this societal recognition often fails to translate into empathy or understanding in the home.

Previous studies have documented that many firefighters voluntarily participate in household and childcare duties after night shifts—even when encouraged to rest by their spouse [12]. However, the current study adds a novel perspective by identifying the “white lie mechanism” as a unique, unintended consequence of protective behavior. By downplaying their work’s emotional and physical toll, firefighters inadvertently undermine opportunities for family members to offer appropriate emotional support. This underexamined mechanism may contribute to weakened familial resilience in coping with cumulative occupational stress.

### 4.5. Guilt-Ridden and Indebted Superman

Firefighters reported profound feelings of guilt related to their inability to participate in important family events because of irregular work schedules. This guilt was compounded by their perception that their family members were making ongoing sacrifices on their behalf—sacrifices they viewed as emotional “and debts” to be repaid. Consequently, many participants engaged in compensatory behaviors such as taking on excessive domestic and childcare responsibilities, often at the expense of their own rest and well-being.

A previous qualitative study involving American firefighters noted that many chose to forgo sleep after night shifts to spend time with their spouses or children [13]. The present study extends these findings by revealing that firefighters’ behaviors are not solely driven by a sense of duty or reciprocity but also by deeply internalized guilt. This guilt compelled participants to abandon personal hobbies, leisure time, and rest to compensate for what they perceived their jobs as a burden on their families.

For instance, statements such as “I gave up hobbies like exercising and always live with guilt,” and “I once dozed off on a park bench while watching my child and did not realize they had fallen” reflect the extreme self-sacrifice many firefighters impose on themselves—even when already physically and emotionally depleted. This extreme self-sacrifice, which extends beyond indebtedness to family and involves abandoning personal life, is a novel finding not discussed in prior research.

### 4.6. Endeavoring to Be Superman at Home as Well

The firefighters in this study actively restructured their post-shift lives to minimize the impact of occupational stress on their families. Strategies include suppressing emotional responses, avoiding discussions about traumatic events, and implementing decontamination routines to reduce exposure to hazardous substances. Furthermore, participants described their efforts to resolve role conflicts by adjusting their household routines and responsibilities.

While some firefighters reported receiving empathy and emotional support when disclosing traumatic experiences to their spouses, others described feeling hurt when they encountered dismissive or judgmental responses. This duality illustrates the fragile nature of emotional disclosure among high-risk occupational families.

Previous studies have shown that empathic listening by spouses may mitigate firefighters’ post-traumatic stress symptoms [28,43]. However, this study qualitatively illustrates, extending prior work, that unsympathetic or invalidating spousal responses may intensify psychological distress and foster emotional isolation. This insight underscores the complexity of interpersonal dynamics in trauma recovery. It highlights the need for psychoeducational interventions and communication-focused support that directly involve the family members of first responders.

Based on the six thematic findings discussed above, we propose integrated intervention strategies to support firefighters’ and their families’ mental health and well-being.

First, psychoeducation for family members is essential to help them recognize irritability, authoritative communication, and hyperarousal not as personality flaws, but as symptoms of PTSD. Reframing these behaviors as trauma-related reactions rather than individual temperaments may reduce interpersonal conflict and promote empathy within the household. Immediate psychological debriefing for firefighters should be provided after emotionally charged incidents, especially those involving child casualties, to create safe spaces for emotional expression and early stress mitigation.

Second, policy-level interventions are needed to reduce caregiver burden and better delineate work and home life boundaries. Guaranteed rest periods of at least eight uninterrupted hours following shift duty could be operationalized through mandatory recovery protocols integrated into national fire service regulations, with compliance monitored via duty logs. Moreover, municipal fire departments could pilot on-site childcare facilities during night shifts or establish formal partnerships with community emergency-care providers, with partial government funding support, to better assist spouses who shoulder the primary responsibilities for household and childcare duties.

Third, formal peer support programs could be embedded into weekly team meetings or annual resilience training modules, ensuring structured participation rather than voluntary attendance, in order to prevent extreme self-sacrifice driven by guilt and emotional indebtedness, which often lead to burnout. Promoting participation in hobby-based clubs and leisure activities may further contribute to psychological recovery and work–life balance.

Finally, scenario-based workshops could be incorporated into existing annual family education sessions, supported by standardized listening-guideline manuals distributed nationwide, to enhance nonviolent communication and active, non-judgmental listening skills among firefighters and their family members. This should be complemented by creating a listening guideline manual for spouses that offers specific strategies for fostering emotional support during trauma disclosure. Given the previous evidence that partners may experience secondary traumatic stress [28,43], tailored counseling programs for family members should also be considered. These initiatives may help to strengthen the family unit as a critical source of psychological resilience for firefighters.

While the clinical and counseling implications of our findings are important, they can also be understood in terms of organizational behavior and management. Work–family conflict is not only an individual or family concern but also a critical organizational issue that affects retention, job performance, and long-term workforce sustainability. In line with the JD–R model, our findings suggest that organizational resources can buffer the negative impact of high job demands on family life. Thus, interventions should include family-supportive supervisory training, flexible scheduling policies, and formal peer-support programs, which are well established in organizational research as strategies that reduce work–family conflict and enhance employee well-being. Our qualitative findings—especially firefighters’ accounts of family strain from irregular shifts, limited family understanding of their workload, and guilt that led to overextension at home—directly ground these organizational recommendations.

Based on the findings discussed above, several limitations of this study should be acknowledged, and suggestions for future research should be made.

First, it focused exclusively on male firefighters in Busan Metropolitan City, which is widely considered the second capital of South Korea. Therefore, the findings may not fully reflect the experiences of firefighters in other regions or those of female firefighters, and gender differences are likely to shape work–family conflict in unique ways. Future studies should include participants from diverse geographical and demographic backgrounds to capture a more comprehensive range of experiences.

Moreover, although contrasting experiences were observed (e.g., supportive versus dismissive spousal responses; negotiated rest routines), systematic analysis of negative cases and subgroup comparisons was limited. Future studies should purposively recruit larger, role-stratified, and family-stage–diverse samples to examine this heterogeneity in greater depth.

Second, there is an urgent need for qualitative studies that incorporate the perspectives of firefighter spouses and family members. To date, no research in South Korea has examined the lived experiences of firefighter spouses in the family context. Just as male firefighters experience unique difficulties at home, their spouses are also likely to face distinctive challenges stemming directly from their husbands’ occupation. Capturing these experiences through the spouses’ own voices is therefore essential. Future investigations should consider direct interviews with spouses or adopt family-centered qualitative or mixed-methods designs. Moreover, intervention studies are needed to develop and evaluate counseling and educational programs aimed at mitigating the effects of secondary traumatic stress among family members.

Third, this study was conducted using telephone-based, cross-sectional, and in-depth interviews, which limited the ability to observe nonverbal cues and precluded causal inferences, potentially reducing some qualitative depth. Future studies should consider face-to-face interviews or longitudinal research designs to track psychological and emotional changes accurately.

Furthermore, because only married firefighters who self-reported family-related difficulties were included, the sample may not fully represent the broader firefighter population, including unmarried firefighters or those who did not perceive such challenges. This selection criterion may therefore limit the generalizability of the findings. Future studies should expand recruitment to more diverse groups to capture variations in experiences across different demographic and family contexts.

## 5. Conclusions

This study employed in-depth individual interviews and a phenomenological approach to explore firefighters’ challenges in their families. These findings indicate that mental health difficulties resulting from job-related trauma and occupational stress can significantly disrupt family dynamics, weakening the supportive role of the home environment and, in some cases, turning it into a source of conflict. These qualitative insights offer a valuable empirical foundation for future research, policy initiatives, and the development of targeted interventions to support firefighters and their families.

These results underscore the importance of educational and counseling programs for firefighter families, couple-based communication training, and emotional support interventions. Furthermore, family-friendly policies that consider the demands of irregular shifts are essential. Collectively, these efforts may enhance marital satisfaction, reduce work–family conflict, and ultimately reinforce the family unit as a critical source of psychological resilience for firefighters. Such improvements are vital for the mental well-being of individual firefighters and instrumental in promoting operational safety, public trust, and broader societal resilience.

While this study provides important insights, the findings should be interpreted with caution given its focus on a single city and a predominantly married male sample within a specific cultural context. Future research should involve spouses, explore gender differences, and adopt longitudinal designs.

## Figures and Tables

**Table 1:** Themes and subthemes identified from the lived experiences of male firefighters regarding family-related difficulties.

Themes	Subthemes
Acting as an emergency commander at home	Heightened arousal Sleep disturbances affecting the spouse Authoritative communication leading to conflict
Reinterpreting traumatic experiences after marriage	Irritability or withdrawal at home after traumatic incidents Excessive safety warnings toward family Increased appreciation for family Greater emotional involvement in incidents after having children
Physical and emotional exhaustion due to irregular work schedules	Inadequate rest after night shifts Anxiety about family’s safety during night shifts Heightened conflict with spouse over childcare Sacrificing personal plans due to emergency mobilization
National heroes misunderstood by their families	Families consider standby time as rest time Lack of recognition for post-night-shift recovery time Complaints about missing important family events
Guilt-ridden and indebted Superman	Guilt for missing important family moments Feeling indebted to family members who endure a lot Guilt toward family members who worry about their safety Overextending themselves to compensate their families
Endeavoring to be Superman at home as well	Self-control to prevent firefighter traits from affecting family life Concern about passing ‘bad things’ to the family Sharing trauma with their spouse Adjusting daily life to maintain work–family balance Increased caution to reassure the family

## Data Availability

The datasets generated and/or analyzed during the current study are not publicly available due to the sensitive and potentially identifying nature of qualitative interview data. De-identified excerpts may be available from the corresponding author upon reasonable request and with IRB approval.

## Abbreviation

The following abbreviation is used in this manuscript:

PTSD	Post-traumatic stress disorder
